# Self-Selected Walking Cadence after 16-Week Light-Intensity Physical Activity Intervention for Older Cancer Survivors

**DOI:** 10.3390/ijerph19084768

**Published:** 2022-04-14

**Authors:** Elizabeth M. Harding, Ann L. Gibson, Huining Kang, Micah N. Zuhl, Harsh Sharma, Cindy K. Blair

**Affiliations:** 1 Department of Rehabilitation and Movement Science, University of Vermont, Burlington, VT 05405, USA; elizabeth.harding@med.uvm.edu; 2 Department of Health, Exercise, and Sports Sciences, University of New Mexico, Albuquerque, NM 87131, USA; alg@unm.edu; 3 Department of Internal Medicine, University of New Mexico, Albuquerque, NM 87131, USA; hukang@salud.unm.edu (H.K.); hrsharma@salud.unm.edu (H.S.); 4 Comprehensive Cancer Center, University of New Mexico, Albuquerque, NM 87131, USA; 5 Exercise Science Division, School of Health Sciences, Central Michigan University, Mt Pleasant, MI 48859, USA; zuhl1m@cmich.edu

**Keywords:** ActivPAL, wearable technology, cadence bands, cancer survivors, peak cadence, average cadence

## Abstract

In this secondary analysis of a light-intensity physical activity intervention, we hypothesized that older cancer survivors would self-select a faster walking cadence to meet their daily step goals. Average steps/day and free-living walking cadence were measured in 41 participants (age 69 ± 3.1 years) with an ActivPAL monitor worn 7 days pre- and post-intervention. Besides peak and average walking cadence, stepping patterns associated with ambulatory intensity were sorted in cadence bands of 20 steps/min from 40–59 (incidental movement) to ≥120 steps/min (fast locomotor movement). Compared to the waitlist Control group (*n* = 17), the Intervention group (*n* = 24) increased their peak 30-min cadence (4.3 vs. 1.9 steps/minute; *p* = 0.03), average 10-min cadence (4.1 vs. −6.6 steps/minute; *p* = 0.04), and average 30-min cadence (5.7 vs. −0.8 steps/minute, *p* = 0.03). Steps taken in cadence bands denoting moderate-intensity physical activity (100–119 steps/min) increased by 478 (interquartile range (IQR): −121 to 1844) compared to decreasing by 92 (IQR: −510 to 181) steps/day for the intervention and Control groups, respectively (*p* < 0.01). Evaluation of free-living walking cadence and patterns of ambulatory behavior can inform future interventions targeting behavior change, especially in those populations most at risk for reduced physical activity and vulnerable to mobility deficits and loss of independence.

## 1. Introduction

There are over 16.9 million cancer survivors in the United States, a number projected to reach close to 20 million over the next ten years [1]. Cancer is highly associated with aging, as 62% of cancer survivors are 65 years and older [1]. Early detection and improved treatment have led to an increase in cancer survivorship, but older cancer survivors are faced with a special set of challenges as many are living with multiple comorbidities in addition to age-related health factors such as deficits in physical function (PF) [2,3]. This may exacerbate the already detrimental effects of cancer and cancer treatments and can have a negative impact on health-related quality of life (QoL) [4]. Furthermore, older adults who attain low levels of daily physical activity (PA) and high levels of sedentary behavior (SB) are more likely to experience mobility deficits and functional limitations than their more active counterparts, the consequences of which increase the risk of long-term disability, morbidity, and mortality [5,6,7,8].

The benefits of regular PA and exercise for attenuating functional decline associated with aging, disuse and disease are well-elucidated. Historically, PA and exercise programs addressing the needs of an older adult population have incorporated structured PA at moderate-to-vigorous intensity, likely due to the strong evidence supporting the relationship between moderate-to-vigorous intensity physical activity (MVPA) and improvements in health-related outcomes [9,10,11,12,13]. These include walking programs and facility-based exercise regimens that incorporate multiple exercise modalities in line with the PA duration and intensity recommendations from the World Health Organization and the American College of Sports Medicine (ACSM) [10,14,15,16,17]. Although there are numerous benefits associated with MVPA, structured exercise at a moderate-to-vigorous intensity may be unrealistic for certain populations. It is possible that interventions promoting a reduction in and replacement of SB with light-intensity physical activity (LPA), such as standing and light-stepping, may bridge the gap between sedentarism and structured exercise programs at the recommended intensity. 

The average number of steps one takes per day is directly associated with cardiometabolic health [18], QoL [19,20,21], bone density [21], and BMI [20,22]. Healthy older adults (≥ 65 years) should aim to achieve 7000–10,000 steps/day [23]. However, an increase of 2000 steps/day over baseline for very sedentary (<5000 steps/day) or functionally limited individuals has resulted in favorable changes in cardiometabolic health and physical function [18]. While steps/day can depict quantity (i.e., volume) of daily PA, measures of cadence can provide greater detail as to the quality of PA (i.e., intensity) and provide a more granular picture of free-living ambulatory behavior. 

Cross-sectional analyses of accelerometry data have shown that older adults and adults with multiple comorbidities spend more time at 0 steps/min (seated, lying) and in cadence bands associated with incidental and sporadic movement (<40 steps/min) and less time ambulating above cadence bands indicative of purposeful movement (≥40 steps/min) compared to their younger and healthier counterparts [24,25,26]. How or at what intensity one ambulates throughout their waking day is of particular importance in SB research due to the distinct physiological mechanisms associated with increased SB and reduced PA. Analyses of time spent in cadence bands of varying intensities may provide some insight as to participant compliance or strategies used to increase PA and decrease SB. Additionally, peak cadence and average cadence can provide insight as to the ‘best natural effort’ and sustained endurance activity, respectively, during typical daily ambulatory activity in a free-living environment [22]. 

While it has been suggested that peak 1-min cadence is a reasonable measure of the highest intensity one is capable of achieving, peak 30-min cadence may be a better representation of the persistence of effort and indication of true behavior change [22,27]. Accelerometer-derived average 30- and 10-min cadence may be considered a measure of endurance as these values represent the highest walking intensity for those given durations. At the time of our study, the ACSM recommended a minimum of 30-min bouts of MVPA most days of the week (150 min/week), which could be accumulated in bouts of 10 or more minutes for individuals with time or health constraints. However, less than 10% of Americans are meeting the minimum MVPA guidelines, and only 2.5% of individuals 60 years and older are meeting these guidelines by accumulating 10-min bouts [28]. A reasonable assumption that an individual is meeting the ACSM PA guidelines as measured by an accelerometer would be an average 30-min cadence of at least 100 steps/min. 

While home-based interventions prescribing structured PA in accordance with current guidelines have been successful at increasing walking cadence in older populations [17,29,30,31], it has yet to be determined if individuals participating in a home-based intervention to replace SB with LPA would self-select to walk at cadences associated with more purposeful movements (≥40 steps/min). There have been few interventions using changes in walking cadence as outcome measures in older and/or special populations. 

The goal of this secondary analysis was to examine changes in walking cadence among older cancer survivors who participated in an intervention to interrupt sedentary behavior with LPA (standing and stepping). Participants in the Intervention group were encouraged to break up sitting time and “move more throughout the day” with the help of the ‘reminder to move’ feature of their activity monitor. Participants were also provided a graduated step goal to incentivize more stepping than standing; however, instructions as to an intensity or minimal bout duration of stepping were not provided.

We hypothesized that interrupting SB with an attainable yet low-intensity step-goal, would prompt individuals to take more purposeful steps at a higher cadence, quantifiable as increases in peak and average cadence values and time spent ambulating at higher intensities. Specifically, we hypothesized that compared to the Control group, the Intervention group would significantly increase: (1) the number of steps per day and time spent in cadence bands indicative of purposeful stepping and of medium-intensity walking; (2) peak 1-min and peak 30-min cadence; and (3) average 10-min and average 30-min cadence. Our rationale was that the reminders to move coupled with the step goal would lead to more participants self-selecting a faster walking speed (cadence) and longer periods of ambulation given their intention to meet their step goal. Exploration into self-selected amount, quality and duration of LPA is novel and could potentially inform future interventions targeting this population. 

## 2. Materials and Methods

### 2.1. Design and Participants

The Move for Your Health (MY Health) Study was a 16-week home-based intervention among older cancer survivors to interrupt sedentary time with LPA. A more detailed outline of all methods and outcomes from the MY Health Study have been published [32]. Research protocols were approved by the University of New Mexico’s Institutional Review Board and written informed consent was received from participants prior to beginning the intervention. 

Fifty-four MY Health Study participants, ages 60 years and older, were recruited from Albuquerque and surrounding communities via the New Mexico Tumor Registry (NMTR) and flyers placed throughout the community. Participants were eligible if they had been diagnosed with cancer and completed primary treatment, owned a smartphone, were able to read and understand English, lived independently, and were able to walk three blocks (~0.25 mi) without the aid of an assistive device. Potential participants were excluded if they worked or volunteered outside the home more than 20 h per week or had any severe impairments or pre-existing medical conditions that prevented them from participating in light-intensity physical activity. 

### 2.2. Instrumentation

The data for the primary outcomes used in these analyses were collected from the ActivPAL3 micro monitor (PAL Technologies Ltd., Glasgow, UK). The ActivPAL3 micro is a small, thin monitor that affixes to the midline of the mid-right thigh with a Tegaderm™ adhesive dressing. The proprietary algorithms of the ActivPAL3 have an excellent correlation (r = 0.96) with direct observation and accurately distinguish between sitting/lying, standing and stepping behaviors [33]. Because of these features, the use of ActivPAL technology has gained recognition as the gold standard for objectively measured SB [34]. During the first clinic visit, investigators demonstrated the monitor application procedure and then observed while the participant applied the activity monitor. Participants were mailed the ActivPAL3 monitor and an instruction packet outlining how to apply the monitor prior to their follow-up clinic visit. On both occasions, participants were asked to wear the monitor for one week (seven consecutive days) and were provided with monitor logs for tracking their self-reported sleep/wake times and monitor removal during each week’s worth of wear. Participants were instructed to only remove the monitor if swimming, bathing, or any other activity that would require the monitor to be submerged in water or in the event of skin irritation under or surrounding the Tegaderm™ dressing.

Data were collected pre- and post-intervention at the default sampling rate of 20 Hz and downloaded using the manufacturer’s software. ActivPAL3 summary events files were processed using customized scripts in SAS to assess movement behaviors occurring in 1-min epochs. Participant data were included in the analyses if they wore the monitor for ≥10 h per day for at least 4 days. Sleep and wear time were visually confirmed using ActivPAL proprietary image files and compared against wear-logs from participants. Methods similar to those of Tudor-Locke et al. and Barreira et al. were used to quantify daily time (minutes) and steps accumulated in the following cadence bands: 20–39 (sporadic movement), 40–59 (purposeful steps), 60–79 (slow speed walking), 80–99 (medium speed walking), 100–119 (brisk walking), and >120 steps/min (fast locomotor movements) [27,35]. Additionally, daily peak 1- and 30-min cadence values were calculated according to the methods outlined by Tudor-Locke and Gardner [35,36]. Ten- and 30-min average cadence values were calculated using a 10- and 30-min sliding window, respectively, to find the average of the highest 10 and 30 consecutive minutes. 

### 2.3. Intervention

Participants were randomized to one of three groups, Health Coaching (HC), Tech Support (TS) and Wait-list Control (WC). The TS and HC groups received a Jawbone UP2 activity monitor (Jawbone, San Francisco, CA, USA), educational materials about the negative effects of SB and general suggestions for breaking up sedentary time, and an individual project schedule used during their individual health-coaching and tech-support phone calls with a trained investigator/specialist. Throughout the intervention, the technical support specialist helped TS participants change settings and update their goals through the Jawbone UP2 smartphone app. In addition to technical assistance, participants in the HC group were provided encouragement and suggestions to help motivate them in modifying their sedentary behavior. Individuals randomized to WC received instructions to continue their typical daily physical activity levels for 16-weeks. At week 16, they received a Jawbone activity monitor and an abbreviated form of the intervention during their follow-up clinic visit. 

Participants’ initial daily step goal was determined at week four by adding 1000 steps to their previous week’s step count as determined by the Jawbone UP2 activity monitor. Daily step goals were increased by 500 steps/day every two weeks thereafter until a goal of 3000 steps/day above baseline was achieved at week 12. Participants were instructed to maintain this new step goal for the next four weeks until conclusion of the intervention. The Jawbone Idle Alert was initially set to 1 h at which time a slight vibration of the wrist-worn monitor would inform the participant that they had been sedentary for 60 min. The idle alert setting was reduced to every 45 min from weeks six through nine, and every 30 min thereafter until conclusion of the intervention. 

### 2.4. Other Data Collection

Participants reported their general health status (excellent, very good, good, fair, poor) and completed the Self-Administered Comorbidity Questionnaire (SCQ) to identify any additional chronic conditions, such as heart disease or diabetes, for which they had been diagnosed. The SCQ further assesses whether or not the participant was receiving treatment for the condition(s) and if this caused any limitations in their daily activities [37].

Participants completed the Short Physical Performance Battery (SPPB) at both baseline and follow-up clinic visits. The SPPB is an objective assessment tool used to evaluate lower extremity function in older individuals and has been found to be predictive of fall-risk, loss of independence and mortality [6,38,39,40]. The three areas of assessment are balance, mobility, and gait speed over a distance of 8 feet. The highest score attainable on the SPPB is 12 points, with each assessment having a possible score of 4 points. Higher scores indicate better performance. 

Data regarding cancer diagnosis (type of cancer and age at diagnosis) were obtained from the New Mexico Tumor Registry.

### 2.5. Outcome Measures

Outcome measures assessed at baseline and follow-up (week 16) included changes in peak 1- and peak 30-min cadence, average 10- and 30-min cadence, and change in steps taken in six cadence bands of increasing intensities, from accumulated sporadic movements to fast locomotor movements. Cadence bands associated with ambulatory behavior (specifically stepping) begin at 20 steps/min (sporadic movement) and increase in increments of 20 steps/min, up to a cadence band of ≥120 steps/min. Therefore, the average daily number of steps and the total time spent engaged in ambulatory behavior was determined as the sum of all time spent ambulating at ≥20 steps/min averaged across valid wear days. Peak and average cadence values were also averaged across valid wear days at pre- and post-intervention time points. 

### 2.6. Statistical Analyses

Seven participants from the Intervention groups dropped out prior to their follow-up visit; reasons included personal or family illness (*n* = 2), loss to follow-up (*n* = 2), moved out of state (*n* = 1), inconvenience (*n* = 1), and frustration with technology (*n* = 1). Compared to individuals who completed the study, non-completers were more likely to be female (5/7, 71% vs. 25/47, 53%), have a higher BMI (34.4 kg/m^2^ vs. 29.5 kg/m^2^), and report poor or fair health at baseline (3/7, 43% vs. 5/47, 11%). Six participants had incomplete data from their physical activity monitor (5 Intervention group; 1 Control group; insufficient wear time (*n* = 3); monitor malfunction at baseline or follow-up (*n* = 3)). Given the exploratory nature of this analysis, the data were examined using a per-protocol analysis, in that only the 41 participants with complete pre- and post-intervention ActivPAL3 data have been included. Included participants with complete data had similar characteristics compared to excluded participants with incomplete data (Baseline variables: Age 70.1 vs. 66.7; BMI 29.2 vs. 31.5; SPPB score: 10.9 vs. 11.5). The purpose of this secondary analysis was to examine the self-selected walking cadence among participants receiving the intervention; therefore, the HC and TS groups were combined to form a single group, resulting in an Intervention group of 24 participants and 17 in the WC group. Statistical analyses included independent- and paired-samples t-tests for comparisons of continuous variables between and within groups, respectively. Categorical data were assessed using Fisher’s exact tests. Graphical methods (qq-plots and histograms) and the Shapiro–Wilk test for normality (*p* ≥ 0.05, normal) were used to identify potential outliers and deviations from normality. In the event that assumptions of normality were violated, Wilcoxon rank-sum- (independent samples) and Wilcoxon signed-rank (paired samples) tests were employed. Repeated measures analysis of variance (ANOVA) was used to evaluate group differences regarding number of steps and time spent in cadence bands of increasing intensity. Data are presented as median and interquartile range (IQR; 25th to 75th percentile). 

We conducted post hoc analyses to determine if changes on average or peak cadence values from pre- to post-intervention (outcome) within the Intervention group (exposure) were influenced by lower-extremity physical function (i.e., SPPB score). Analyses of peak- and average-cadence variables were conducted in those participants with greater room for improvement, identified as having an SPPB score ≤ 10 out of a possible 12 points [41]. Older adults with SPPB scores ≤ 10 have a significantly greater risk of mobility disability. Additionally, Spearman’s rank correlation coefficient was used to examine the correlation between baseline measures of and change in PA (steps/day) to gather insight as to whether baseline or change in PA may have influenced change in peak and average cadence outcomes. All statistical analyses were conducted using SAS software (Version 9.4, Copyright 2002–2012 by SAS Institute Inc., Cary, NC, USA). Significance was considered at *p* < 0.05.

## 3. Results

Characteristics of the 41 MY Health study participants included in these analyses are presented in Table 1. There were no significant differences at baseline between the Intervention and Control groups. Over half (56.1%) of the study participants were female, and the mean age of participants at baseline was 70.1 (±4.4) years. The mean age of study participants at cancer diagnosis was 65.6 (±4.3 years), with breast and prostate cancer reported most frequently, affecting 34.2% and 29.3% of the study population, respectively. The majority of participants (53.7%) self-reported to be in “very good to excellent” health, yet 61% of participants had three or more co-existing medical conditions at baseline. Eighty percent of study participants were classified as overweight to obese (BMI ≥ 25.00 kg/m^2^).

### 3.1. Quantity of Ambulatory Behavior

On average, the Intervention group participants wore the activPAL monitor for 6.8 (SD = 0.4) days, 14.5 (SD = 0.8) hours/day and the Control group participants wore the monitor for 6.7 days (SD = 0.5), 14.6 (SD = 1.0) hours/day. A summary of the average number of steps per day measured at pre- and post-intervention time points is presented in Table 2. Increases in average daily steps from pre- to post-intervention were significant within the Intervention group (*p* = 0.02). The median increase in steps per day was 976 (IQR: −388–3532), representing a 14% increase on average daily steps within the Intervention group compared to a 2% increase (Median = 354; IQR: −658–1300 steps) observed in the Control group.

### 3.2. Quality of Ambulatory Behavior

#### 3.2.1. Purposeful Stepping and Medium-Intensity Walking

Changes in the time spent and steps taken in cadence bands indicative of purposeful movement (≥40 steps/min) and medium intensity walking (≥80 steps/min) are presented in Table 3. The changes in the number of steps taken at cadences of 40 steps/min or above (954 vs. 327 for Intervention vs. Control groups) indicate that the majority (92–98%) of the additional steps per day were performed at a purposeful or higher cadence. The changes in the number of steps taken at a medium-intensity walking cadence or higher (≥80 steps/min) increased by 71% in the Intervention group (*p* < 0.01) compared to 18% in the Control group. However, there was not enough evidence supporting a between-group difference (*p* = 0.08). 

Within the Intervention group, the time spent at cadences ≥ 40 steps/min increased from 90.2 to 102.3 min per day. However, the proportion of time spent at cadences ≥ 40 steps/m relative to the total time spent in ambulatory activity was 94% for both pre- and post-intervention time points (95.6 to 108.7 min total ambulation time pre- to post-intervention), indicating no change within the Intervention group. The Control group also increased time spent in cadences ≥ 40 steps/min from 97.4 to 99.3 min per day, suggesting an increase of 4% when considered relative to total time spent engaged in ambulatory activity (109.0 to 106.3 min total ambulation time pre- to post-intervention). When evaluating time spent engaged in medium intensity or higher walking (≥80 steps/min) relative to the total time spent ambulating, the Intervention group exhibited an 8% increase from pre- to post-intervention versus a 3% increase observed in the Control group. However, within- (pre- to post-intervention) and between-group differences were not significant when time spent at cadences ≥40 and ≥80 steps/min were evaluated as respective proportions of total time spent ambulating (*p* > 0.05). 

#### 3.2.2. Distribution of Steps across Cadence Bands

The average number of steps taken within cadence bands associated with increasing intensities of ambulatory behavior are included in Table 4. The Intervention group increased the amount of time spent in the cadence band associated with brisk walking (aka moderate-intensity physical activity). This is in contrast to the Control group, which increased the time spent engaged in slow- and medium-intensity walking, but decreased the amount of time spent engaged in brisk walking. Results from the repeated measures ANOVA on the six cadence bands identified a significant interaction of the intervention on cadence bands for time spent stepping (*p* < 0.001). Post hoc analyses found a between-group significance for cadences between 100–119 steps/min. No other between-group differences were noted for changes in time spent and steps taken within cadence bands. Additional inquiry into time spent at cadences between 100 and 119 steps/min revealed that, on average, nine participants in the Intervention group (~38%) increased the time spent in moderate intensity PA by ≥10 min compared to zero participants in the Control group (Fisher’s exact, *p* = 0.01; data not shown).

#### 3.2.3. Peak Cadence

Pre- and post-intervention values and change in peak and average cadence are displayed in Table 5. There was a modest increase of 3.8 steps/min (IQR: −5.8–7.7) in peak 1-min cadence for the Intervention group compared to a decrease of 0.87 (IQR: −5.8–7.7) steps/min in the Control group. Between- and within- group comparisons did not reach significance (*p* > 0.05). Peak 30-min cadence increased in the Intervention group by 4.3 steps/min compared to an increase of 1.9 steps/min in the Control group (*p* = 0.03). The median increase of 4.3 (IQR: −0.96–16.8) steps/min translates to a persistence of effort approximating an additional 130 steps over 30 min. 

#### 3.2.4. Average Cadence

Between-group differences in the change in average 30- and 10-min cadence values were observed (*p* < 0.05). Over the highest average 30-min cadence, the Intervention group increased their steps by approximately 171 (stepping rate increase of 5.7 steps/min) compared to a 25 step decrease in the Control group (*p* = 0.03). Likewise, the Intervention group increased their average 10-min cadence by 4.1 steps/min (an average increase of 41 steps over 10 min) while the Control group had a decrease of 6.6 steps/min (average of 66 steps over 10 min; between group *p*-value = 0.04).

### 3.3. Post Hoc Analyses

#### 3.3.1. Room for Improvement—Potential Ceiling Effect of Baseline Physical Performance

**Figure 1:**
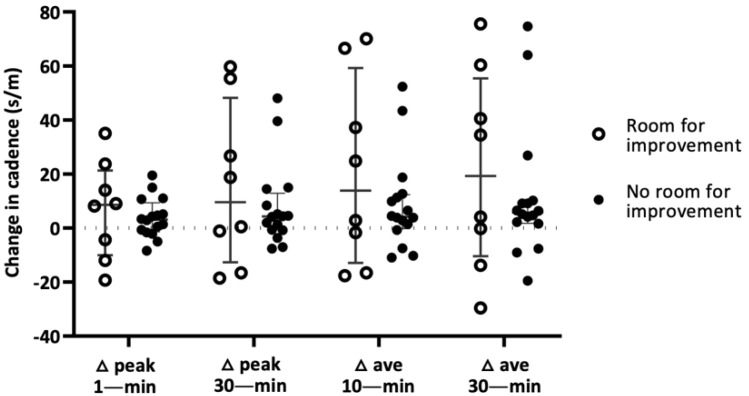
Changes in cadence values based on those with or without room for improvement according to objective physical function scores at baseline. Vertical bars represent the median (inner horizontal dash) and the interquartile range (outer horizontal dashes). Open circles represent individuals with room for improvement as determined by SPPB score of ≤10. Solid circles represent individuals with less room for improvement based on SPPB score of >10. Δ: change. s/m: steps per minute.

To explore potential ceiling effects, the influence of the intervention on the changes in peak- and average-cadence variables were reevaluated according to baseline physical performance (Figure 1). Eight of the 24 Intervention group participants had SPPB scores ≤ 10, resulting in a ‘room for improvement’ classification, while 16 were designated as having ‘less room for improvement’ (>10 of 12 total). Median peak- and average-cadence values (steps/minute) increased in both subsets of the Intervention group, and appeared to be greater among those with room for improvement. However, results should be interpreted with caution, as there is insufficient data to detect any but the largest differences.

#### 3.3.2. Influence of Baseline and Change in Average Daily Steps on Change in Peak and Average Cadence

**Figure 2:**
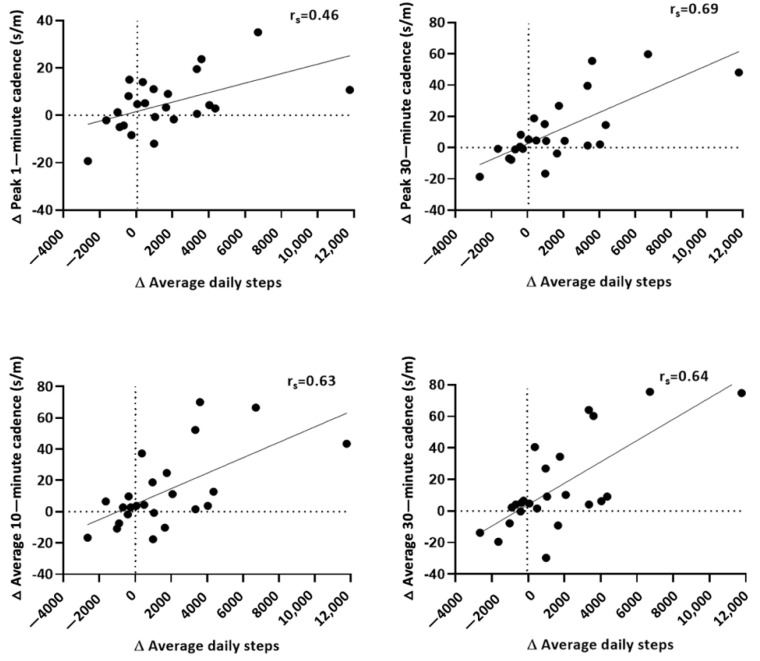
Correlation between change in average daily steps and change in cadence values (upper left: peak 1—min cadence; upper right: peak 30—min cadence; lower left: average 10—min cadence; lower right: average 30—min cadence). Solid circles represent Intervention group participants. r_s_: Spearman’s rank correlation defined as: Weak r_s_: ±(0.00 to <0.40); Moderate r_s_ = ±(0.4 to <0.6); Strong r_s_: 0.6 to <0.8); s/m = steps/min.

Individuals with the lowest average daily steps at baseline had the largest improvement in peak and average cadence (Spearman’s rank correlation: r_s_ = −0.43 to −0.60; data not shown). There was a moderate correlation (r_s_ = 0.46) between the change in average daily steps and change in peak 1-min cadence (Figure 2). The correlations (r_s_ = 0.63 to 0.69) were somewhat stronger between change in average daily steps and changes in peak 30-min and average 10- and 30-min cadence values, suggesting that individuals who had a greater change in average daily steps also increased their free-living walking speed. 

## 4. Discussion

This is the first study to investigate cadence metrics in a population of older cancer survivors with comorbidities, and one of the few to evaluate cadence in a free-living population. While there was only a modest improvement in the quantity of ambulatory behavior (daily steps) among the Intervention group compared to the Control group, there was evidence of a significant improvement in the quality of ambulatory behavior. This was evidenced by improvement in peak (30-min) and average (both 10- and 30-min) cadence, as well as number of steps taken in the brisk walking (moderate physical activity) cadence band. 

Peak 1-min cadence represents the highest number of steps/min in a single day and may represent one’s ‘best natural effort’, or rather the free-living walking cadence of which an individual is capable. Peak-1 min cadence is highly dependent on age, physical activity level (i.e., steps/day), physical function, and body mass index (BMI) [18,42]. Analyses of cross-sectional data have indicated that, on average, peak 1-min cadence in healthy adults (<60 years of age) is approximately 100 steps/min and can be lower (94.2 to 81.5 steps/min) in older (>70 years), or relatively unhealthy adults [43]. While 80% of the MY Health Study participants included in these analyses were classified as overweight to obese (BMI ≥ 25 kg/m^2^), objective measures of PF indicated an above average to high level of physical functioning for the sample. Slow to very slow walking cadences of 68 to 88 steps/min have been found among sedentary (<5000 steps/day) to very sedentary (≤2500 steps/day) individuals, often concomitant with advanced age and chronic illness [42,44,45]. However, over 70% of MY Health Study participants were classified pre-intervention as being low-active (5000–7499 steps/day) to highly active (≥12,000 steps/day). Therefore, it was not surprising that median peak cadence values for both groups exceeded 100 steps/min at baseline and that there were no significant differences for peak 1-min cadence between groups after the intervention. This may be attributed to the already high peak 1-min cadence exhibited among participants at baseline, potentially leaving very little room to increase stepping rate within such a brief (1-min) period. 

In contrast to the peak 1-min cadence, peak 30-min cadence as a metric of ‘persistence of effort’ has been suggested to be more characteristic of true behavior change compared to the peak 1-min cadence [27]. Our finding of significant between-group differences for the change in peak 30-min supports findings from interventions using walking behavior (or increased steps/day) as a means to increase PA [27,46]. Although there were no specific guidelines regarding intensity or duration of physical activity in the MY Health Study, self-selection of a faster cadence associated with participant intention to move (i.e., increase steps/day) were evident. 

Significant increases between groups were noted for average 10- and 30-min cadence variables even though no explicit suggestions were given to the participants that they achieve their daily step goal in 10 or 30 consecutive minutes. However, participants were encouraged to increase their daily steps by 3000 above baseline over the course of the 16-week intervention. The motivation to achieve 3000 steps above baseline, coupled with the relatively high pre-intervention daily step count suggests that many participants in the MY Health study self-selected a walking cadence between 100 and 119 steps/min. This implies that some individuals in the Intervention group achieved 30 min of MPA most days of the week (100 steps/min times 30 min = 3000 steps), without being explicitly coached to do so. However, if this were the case for the majority of Intervention participants, then their average 30-min cadence post-intervention should approach 100 steps/min, but it did not. Nevertheless, it nearly doubled from pre- to post-intervention (from median = 30.6; IQR: 25.2–62.3 to median = 60.9; IQR 31.4–93.7 steps/min). This overall change in average cadence among the Intervention group is an exciting finding, as it indicates an increase in endurance, especially in those participants with lower physical function at baseline. While investigation as to whether the increase in endurance was due to an increase in leg strength is beyond the scope of this secondary analysis, it is an area for future study. Interventions utilizing accelerometers to measure changes in cadence variables are still quite novel and future investigations exploring increases in free-living cadence metrics are warranted. 

Given that some clinical populations or those with reduced physical capacity may find long bouts of continuous walking difficult or unfeasible, it was important to investigate between-group differences in step accumulation in continuous 10-min bouts. The Intervention group increased their average 10-min cadence by approximately 4 steps/min. Ten minutes was selected as it was the minimum single bout duration ACSM recommended (at the time of our study) for accumulating one’s weekly dose of MVPA (150 min/week or 30 min most days of the week). However, shorter bouts (<10 min) may still offer cardio protective benefits and have a positive impact on BMI, waist circumference, triglycerides, and cholesterol as long as the recommended goal of ≥150 min/week of MVPA is met [47]. Still, more longitudinal and randomized controlled trials are needed to more fully examine the effects of shorter bouts (<10 min) of PA on health outcomes, independent of other lifestyle factors such as physical fitness and dietary habits. 

### 4.1. Influence of Baseline Physical Function on Cadence Outcomes

We separated the Intervention group into those with room for improvement and those with little to no room for improvement based on an SPPB score of ≤10 (room for improvement). Although not statistically powered to see all but the largest changes, the room for improvement group showed relatively large increases in median peak- and average-cadence variables compared to the little to no room for improvement group. There was a large amount of variability among the participants within the Intervention group, especially the eight participants with room for improvement. This could certainly obscure any within-group differences, but as noted in the results, there was a two- to three-fold increase in peak- and average-cadence values observed in those individuals with baseline SPPB ≤ 10. While this could indicate a potentially meaningful improvement in cadence variables, further investigation is warranted. It would also be interesting to know more about the individuals within the Intervention group that failed to demonstrate increases in peak and average cadence despite having room for improvement. It is possible that a participant had a substantial change in health status over the course of the 16-week intervention, or perhaps non-compliance was a factor. These factors could certainly influence our findings, and if possible, should be examined further, if for no reason other than to inform future interventions. 

### 4.2. Influence of Baseline and Change in Physical Activity on Cadence Outcomes

There were moderate inverse correlations between baseline physical activity (steps/day) and changes in peak cadence variables, indicating that participants with fewer steps/day at baseline improved more during the intervention period than did their more physically active counterparts. These findings point to a potential ceiling effect limiting the ability of individuals having a high number of steps/day at baseline to significantly increase their peak and average cadences. It is plausible that some participants in the sample were more habitually physically active, likely to walk for leisure/fitness, and ambulating at their preferred pace at baseline than were their more sedentary counterparts. However, the systematically decreasing idle alert periods and increasing steps/day targets of the intervention successfully improved peak- and average-cadence values among participants with reduced baseline PA and is a viable prescription for increasing daily PA for sedentary (<5000 steps/day) older adults similar to those in this sample. 

The moderate to strong correlations between change in average daily steps and changes in peak- and average-cadence variables suggest that those who increased their daily steps took those steps at a faster cadence. Cross-sectional analyses and interventions targeting clinical populations have demonstrated a strong relationship between average daily step count and habitual cadence. Therefore, our results are not surprising. However, further investigation as to motivational factors could provide additional insights as to the nature of these increases and the causal influence of daily steps on free-living walking cadence. 

### 4.3. Limitations

Although carefully executed, these analyses are not without limitations. This is a secondary analysis of an intervention where outcomes related to free-living walking cadence were not considered in the original study design. The MY Health study was a feasibility study and thus was not statistically powered to detect small differences between the two groups. A potential limitation is the inclusion of individuals with high physical functioning and physical activity levels at baseline, thus limiting room for improvement. Nevertheless, improvements in endurance and persistence of effort were observed. Additionally, these results may not generalize to older cancer survivors with very low physical functioning, especially those with or at high risk for mobility disability. Another limitation is the lack of information on whether participants met their bi-weekly step goals. We were unable to download data from the Jawbone tracker website for all participants, as the company went out of business in 2017. 

### 4.4. Future Directions

The usefulness of the time-stamp feature of most modern accelerometers affords the opportunity to investigate patterns and quality of ambulatory behavior to better detail habitual or malleable ambulatory activities beyond the typical quantification of steps/day or minutes of MVPA. However, an important consideration when evaluating accelerometric data is that the environmental context for which the PA is taking place is lacking. Additional sources (GPS, camera, or ecological momentary assessment) will be beneficial in capturing this information. While several of the hypotheses regarding peak- and average-cadence metrics were supported, current analyses do not identify specific combinations of intensities used, or to what extent participants self-selected to achieve their daily step goal. Further investigation as to temporal patterns throughout daily waking hours could provide additional insight as to strategies used by participants when given a simple goal in which there are multiple ways to achieve that goal.

## 5. Conclusions

In this 16-week intervention designed to replace SB with LPA, there was a substantial number of participants who chose to walk at medium to brisk intensity despite no stipulations on minimum bout duration or minimum walking intensity. Increases in peak 30-min cadence resulted in gains of persistence of ambulatory effort and demonstrated that stabilization of a faster habitual walking speed is possible for participants similar to those in this study. Although the goal of the intervention was to accumulate steps over the course of an entire day, participants, on average, self-selected to do so in longer bouts at higher intensities. Evaluation of free-living walking cadence and patterns of ambulatory behavior can inform future interventions targeting behavior change, especially in those populations most at risk for reduced physical activity and vulnerable to mobility deficits and loss of independence.

## Figures and Tables

**Table 1:** Baseline participant characteristics.

	Intervention	Control
Characteristics	*n =* 24	*n* = 17
Female; *n*(%)	14 (58.3)	9 (52.9)
Age (years): mean ± SD	69.6 (3.4)	70.8 (5.4)
Height (cm): mean ± SD	164.9 (11.4)	168.3 (11.0)
Weight (kg): mean ± SD	79.0 (16.2)	83.6 (16.8)
BMI (kg/m^2^): mean ± SD	29.0 (4.7)	29.5 (5.3)
Cancer type		
Breast; *n*(%)	10 (41.7)	4 (23.5)
Prostate; *n*(%)	6 (25.0)	6 (35.3)
Other; *n*(%)	8 (33.3)	7 (41.2)
Age at diagnosis (years): mean ± SD	65.2 (3.7)	66.2 (5.1)
Health status (self-report)		
Very good/excellent; *n*(%)	13 (54.2)	9 (52.9)
Good; *n*(%)	9 (37.5)	7 (41.2)
Fair/poor; *n*(%)	2 (8.3)	1 (5.9)
Chronic conditions (comorbidities) ≥ 3	8 (33.3)	7 (41.2)
Race/ethnicity		
White (Non-Hispanic); *n*(%)	19 (79.2)	13 (76.5)
Hispanic; *n*(%)	5 (20.8)	4 (23.5)
Marital Status		
Married or living in marriage-like relationship; *n*(%)	17 (70.8)	14 (82.4)
Not Married (single, divorced, widowed); *n*(%)	7 (29.2)	3 (17.7)
Education		
Less than high school or high school graduate; *n*(%)	3 (12.5)	3 (17.6)
Post high-school training or some college; *n*(%)	6 (25.0)	5 (29.4)
College degree or higher; *n*(%)	15 (62.5)	9 (53.0)
Annual household income		
≥50,000/year; *n*(%)	12 (50.0)	13 (76.5)
<50,000/year; *n*(%)	10 (41.7)	4 (23.5)
Declined response; *n*(%)	2 (8.3)	0

**Table 2:** Average steps/day of participants before and after the 16-week intervention, representing the quantity of free-living ambulatory behavior.

	Average Steps/day		
	Intervention (*n =* 24)	Control (*n* = 17)	^a^ p
Pre	7378 (4368–8586)	7732 (5748–10,022)	
Post	8423 (6001–11,063)	7890 (6159–9627)	
Δ	976 (−388–3532)	354 (−658–1300)	^a^ P_ic_ = 0.19
^a^ p	^a^ P_i_ = 0.02 *	^a^ P_c_ = 0.61	

Data presented are median (IQR). ^a^ p values obtained using Wilcoxon signed-rank tests for within group comparisons (P_i_ = intervention and P_c_ = control). Change (Δ) in step counts between groups (P_ic_) was assessed using Wilcoxon rank-sum test. * Denotes significance (alpha = 0.05). IQR: interquartile range.

**Table 3:** Quality of ambulatory behavior: purposeful stepping and medium-intensity walking.

Variable	Intervention (*n* = 24)				Control (*n* = 17)				P_ic_
	Pre	Post	Δ	P_i_	Pre	Post	Δ	P_c_	
Steps ≥40 s/m	6860 (4128–8183)	8104 (5669–10,647)	954 (−325–3356)	0.01 *	7141 (5440–9437)	7411 (5861–9217)	327 (−558–1230)	0.71	0.15
Steps ≥80 s/m	4125 (2815–6461)	6123 (3480–8117)	679 (−333–2767)	<0.01 *	4651 (3512–6581)	4706 (4204–7079)	59 (−368–764)	0.89	0.08
Time spent ≥40 s/m (min)	90.2 (54.9–113.0)	102.3 (69.7–131.1)	8.4 (−6.0–27.8)	0.04 *	97.4 (72.6–121.2)	99.3 (76.1–117.9)	6.4 (−10.3–17.2)	0.60	0.35
Time spent ≥80 s/m (min)	60.5 (37.8–77.8)	74.3 (50.8–100.3)	7.8 (−4.0–24.6)	0.02 *	66.5 (49.2–89.5)	67.1 (54.5–90.4)	1.2 (−7.2–10.7)	0.70	0.15

Data presented are median (IQR). Wilcoxon rank-sum test used for between-group comparisons and Wilcoxon signed-rank test used for within-group comparisons. P_i_: *p*-values obtained for within Intervention group comparisons; P_c_: *p*-values obtained for Control group comparisons; P_ic_: *p*-values obtained for between group comparisons of change (Δ) variables. Pre: pre-intervention; post: post-intervention; s/m: steps per minute; IQR: Interquartile range. * Denotes significance (alpha = 0.05).

**Table 4:** Number of steps taken within cadence bands (I = Intervention group; C = Control group).

		Sporadic Movement	Purposeful Steps	Slow Walking	Medium Walking	Brisk Walking	Fast Locomotion
Steps/min		20–39	40–59	60–79	80–99	100–119 *	≥120
I	Pre	327	677	1030	1826	1488	103
		(267–517)	(541–981)	(791–1364)	(1448–2342)	(828–2912)	(45–782)
	Post	346	711	1021	1925	3013	245
		(231–498)	(480–1103)	(776–1601)	(1479–3201)	(1424–4190)	(46–1018)
	Δ	−12	−8	4	−29	478	15
		(−53–31)	(−100–77)	(−161–238)	(−270–470)	(−121–1844)	(−20–362)
C	Pre	351	771	1185	2286	2088	106
		(293–585)	(657–1140)	(996–1580)	(2089–3099)	(1658–2968)	(71–194)
	Post	425	915	1249	2462	1996	121
		(347–557)	(747–1023)	(1039–1630)	(1950–3759)	(1165–2317)	(79–205)
	Δ	40	66	208	271	−92	25
		(−100–113)	(−149–226)	(−176–305)	(−139–868)	(−510–181)	(−27–79)

All participants accumulated steps in each cadence band (Intervention group = 24; Control group = 17). Data presented are median (IQR). Δ: represents change from pre- to post-intervention. Repeated measures ANOVA suggested a significant interaction of group and cadence bands (*p* < 0.001. *: Post hoc tests indicate a significant difference between group for steps taken between 100–119 steps/min (Wilcoxon rank-sum, *p* < 0.01). ANOVA: Analysis of variance; IQR: interquartile range.

**Table 5:** Quality of ambulatory behavior: peak and average cadence.

Variable	Intervention (*n* = 24)				Control (*n* = 17)				P_ic_
	Pre	Post	Δ	P_i_	Pre	Post	Δ	P_c_	
Peak 1-min (s/m)	100.8 (90.3–113.0)	108.2 (100.5–115.7)	3.8 (−1.9–10.9)	0.06	103.0 (89.7–110.7)	99.7 (96.7–110.8)	−0.9 (−5.8–7.7)	0.86	0.18
Peak 30-min (s/m)	61.7 (54.2–85.2)	78.5 (61.9–103.2)	4.3 (−0.96–16.8)	0.03 *	63.7 (59.3–87.9)	65.6 (61.3–83.9)	1.9 (−4.4–3.9)	0.82	0.03 *
Average 30-min (s/m)	30.6 (25.2–62.3)	60.9 (31.4–93.7)	5.7 (0.8–30.7)	0.01 *	41.9 (30.5–74.6)	41.7 (29.7–69.7)	−0.8 (−6.9–5.0)	0.61	0.03 *
Average 10-min (s/m)	50.3 (42.5–81.7)	73.7 (50.6–106.0)	4.1 (−1.3–21.8)	0.02 *	61.9 (51.0–87.1)	57.9 (48.0–82.6)	−6.6 (−10.0–5.4)	0.38	0.04 *

Data presented are median (IQR). Wilcoxon rank-sum test used for between-group comparisons and Wilcoxon signed-rank test used for within-group comparisons. P_i_: *p*-values obtained for within Intervention group comparisons; P_c_: *p*-values obtained for Control group comparisons; P_ic_: *p*-values obtained for between group comparisons of change (Δ) variables. Pre: pre-intervention; post: post-intervention; s/m: steps per minute; IQR: Interquartile range. * Denotes significance (alpha = 0.05).

## Data Availability

Aggregate data may be available for research purpose upon reasonable request to the corresponding author.

## Trial Registration

ClinicalTrials.gov NCT03632694; https://clinicaltrials.gov/ct2/show/NCT03632694 (accessed on 4 March 2022).
